# Dispensations of benzodiazepines, z-hypnotics, and gabapentinoids to patients receiving opioid agonist therapy; a prospective cohort study in Norway from 2013 to 2017

**DOI:** 10.1186/s12913-020-05195-5

**Published:** 2020-04-25

**Authors:** Jørn Henrik Vold, Svetlana Skurtveit, Christer Aas, Fatemeh Chalabianloo, Pia Synnøve Kloster, Kjell Arne Johansson, Lars Thore Fadnes

**Affiliations:** 1 Department of Addiction Medicine, Haukeland University Hospital, Bergen, Norway; 2 Department of Global Public Health and Primary Care, University of Bergen, Bergen, Norway; 3 Department of Mental Disorders, Norwegian Institute of Public Health, Oslo, Norway; 4 Norwegian Centre for Addiction Research, University of Oslo, Oslo, Norway

**Keywords:** Opioid substitution treatment, Benzodiazepines, Zopiclone, Zolpidem, Pregabalin, Gabapentin, Opioids, Drug prescriptions

## Abstract

**Background:**

Dispensations of benzodiazepines, z-hypnotics, and gabapentinoids to patients on opioid agonist therapy (OAT) are common and have pros and cons. The objectives of the current study are to define the dispensation rates of these potentially addictive drugs, and whether the number and the mean daily doses of dispensed OAT opioids and discontinuing OAT, are associated with being dispensed benzodiazepines, z-hypnotics and gabapentinoids among patients on OAT in Norway in the period 2013 to 2017.

**Methods:**

Information about all dispensed opioids, benzodiazepines, z-hypnotics and gabapentinoids were recorded from the Norwegian Prescription Database (NorPD). A total of 10,371 OAT patients were included in the study period. The dispensation rates were defined as the number of patients who were dispensed at least one of the potentially addictive drugs divided among the number of patients who have dispensed an OAT opioid per calendar year. Mean daily doses were calculated, and for benzodiazepines and z-hypnotics, stated in diazepam equivalents. The association between dispensed potentially addictive drugs, and the number and the type of dispensed OAT opioids were calculated by using logistic regression models.

**Results:**

Half of the OAT patients received at least one dispensation of a benzodiazepine or z-hypnotic, and 11% were dispensed at least a gabapentinoid in 2017. For dispensed benzodiazepines or z-hypnotics, the mean daily dose was reduced from 21 mg (95% confidence interval (CI): 20–23) diazepam equivalents in 2013 to 17 mg (95% CI: 16–17) in 2017. The mean daily dose of pregabalin increased from 365 mg (95% CI: 309–421) in 2013 to 386 mg (95% CI: 349–423) in 2017. Being dispensed a gabapentinoid (adjusted odds ratio (aOR) = 2.5, 95% CI: 2.1–3.0) or a non-OAT opioid (aOR = 3.0, 95% CI: 2.6–3.5) was associated with being dispensed a benzodiazepine or z-hypnotic. Discontinuing OAT did not affect the number of dispensations and the doses of potentially addictive drugs.

**Conclusion:**

The dispensation rates of potentially addictive drugs are high in the OAT population. Treatment indications, as well as requirements for prescription authority, need to be debated and made explicit. Randomized controlled trials evaluating the benefits and risks of such co-prescription are required.

## Background

Dispensations of benzodiazepines, z-hypnotics, and gabapentinoids to patients on opioid agonist therapy (OAT) have pros and cons. These drugs may increase the mortality among patients on OAT and are associated with criminality, psychosocial problems, and injecting drug use [1, 2]. The use of several potentially addictive drugs is particularly challenging in an OAT program aimed at reducing mortality and injecting drug use and achieve rehabilitation among marginalized and comorbid patients with opioid addiction [3–8]. However, dispensations of benzodiazepines among highly comorbid patients with polydrug use may decrease the mortality if such dispensations make patients abstinent of illegal drugs, and they are followed up strictly by health professionals [2].

The proportion of OAT patients who were dispensed benzodiazepines or z-hypnotics varies across countries. In the United Kingdom, the proportion of OAT patients who were dispensed benzodiazepines and z-hypnotics was 42 and 20%, respectively, between 1998 and 2014 [9]. In Sweden, 32% of OAT patients were dispensed a benzodiazepine, and 41% received a z-hypnotic in the period from 2005 to 2012 [1]. Further, the proportion of Norwegian OAT patients who were dispensed benzodiazepine was 40% in 2005. Regulations of OAT are a typical reason for differences in the proportion of patients who are dispensed, potentially addictive drugs between countries [10]. In some countries, OAT has been a subject for strict regulation, implying that OAT is stopped if used potentially addictive drugs concomitantly [11–13]. Others, e.g., Norway, such discontinuation of OAT in case of benzodiazepine, and z-hypnotic use has become less common compared to during the early 2000s [14, 15].

The knowledge of gabapentinoid use among patients on OAT is lacking. However, for the last couple of years, gabapentinoids, particularly pregabalin, are consumed significantly more often among patients with opioid addiction compared to patients with other drug addictions [16]. Withdrawal symptoms are described if dose reduction or discontinuation [17–20], while using high-dosed gabapentinoids may cause euphoric effects, sedation, hallucinations, dissociation, conspicuous behavior, and the reinforced effect of OAT opioids [8, 16]. Dispensing gabapentinoids among patients with opioid addiction not necessarily indicated misuse or addiction. However, it is worrying if adverse side effects occur when combining with highly potent opioids with OAT opioids [16].

In Norway, opioid agonist therapy has been applied increasingly as an available treatment approach for opioid addiction [15]. In 2017, about 7500 patients received OAT opioids, and the majority received their OAT opioids at least once per week from pharmacies. A decreasing proportion of patients discontinued OAT during the past few years, and in 2017, 681 patients, including those who died, left the treatment [15]. Although, little is known about dispensations of benzodiazepines and z-hypnotics and gabapentinoids among patients on OAT. Thus, this observational study aims to investigate the dispensation rates and doses of these potentially addictive drugs among patients who were dispensed at least one OAT opioids in Norway in the period 2013 to 2017 and those who discontinued OAT. This study has three objectives:

1. To define dispensation rates and mean daily dose of benzodiazepines, z-hypnotics, and gabapentinoids per calendar year.
2. To assess the association between OAT opioids in terms of the number of dispensations and mean daily dose of OAT opioids, and whether patients are dispensed a benzodiazepine, z- hypnotic, or gabapentinoid, or not per calendar year.
3. To evaluate whether discontinuations of OAT affect the number of dispensations and the mean daily dose of dispensed benzodiazepines and z-hypnotics, or gabapentinoids.

## Methods

### Data source

All data were register data and were drawn from the Norwegian Prescription Database (NorPD) (www.norpd.no). From January 1, 2004, all pharmacies in Norway are obliged by law to submit all the dispensed drugs data electronically to the Norwegian Institute of Public Health. NorPD contains information of all dispensed drugs, including reimbursements, dispensed from pharmacies in Norway, except for dispensations dispensed during hospitalizations or at outpatient clinics. Anatomical Therapeutic Chemical (ATC) classification system was employed according to the determination by the World Health Organization (WHO) collaborating centre for drug statistics per October 2018 [21]. NorPD data were collected from January 1, 2013, to March 31, 2018, in this study. The STROBE checklist was applied in the preparation of the study (Additional file 1).

### Study population

All patients above 18 years of age who received at least one dispensation of an OAT opioid, including methadone, levomethadone, buprenorphine, and buprenorphine-naloxone, from January 1, 2013, to December 31, 2017, were included. The patients using methadone tablets to achieve pain relief in palliative care were excluded by identifying those who only were dispensed methadone tablets without any dispensations of other OAT opioids or methadone mixture in the period from January 1, 2004, to December 31, 2017. Discontinuing OAT was defined as all patients who had the last dispensation of an OAT opioid in the inclusion period from January 1, 2017, to September 30, 2017, and then, no dispensations until the end of the collected NorPD data on March 31, 2018.

### Analysis strategy and statistical analyses

#### Definitions of opioids, benzodiazepines, z-hypnotics, gabapentinoids, age, the number of dispensations, mean daily doses, and the type of dispensed OAT opioid

Opioid agonist therapy opioids, non-OAT opioids, and gabapentinoids, including gabapentin and pregabalin, were defined according to their ATC codes (Additional file 2). Benzodiazepines and z-hypnotics were defined as all benzodiazepines and z-hypnotics that have or have had marketing authorizations in Norway during the study period, including alprazolam, clonazepam, diazepam, flunitrazepam, nitrazepam, oxazepam, zolpidem, and zopiclone. Dispensations of benzodiazepines and z-hypnotics were pooled.

All included OAT patients were categorized into age groups per calendar year. The age groups were ≤ 25, 26–35, 36–45, 46–55, and ≥ 56. The number of dispensations was defined as all dispensations of a drug per calendar year. Further, the type of OAT opioid was defined as the last type of opioid that was dispensed per year. The mean daily doses of benzodiazepines, z-hypnotics, and gabapentinoids per year were calculated by using daily defined doses (DDD) stated in NorPD defined by the WHOs standards [21]. For benzodiazepines and z-hypnotics, dispensed DDDs of each benzodiazepine or z-hypnotic were summarized per year and converted to milligrams. For each benzodiazepine or z-hypnotic, the doses per year (in milligrams) were converted to diazepam equivalents, according to a benzodiazepine and z-hypnotic equipotency table stated in the Norwegian national guidelines for addictive drugs (Additional file 3) and WHOs standards [21, 22]. The total doses of each benzodiazepine or z-hypnotic were summarized (in diazepam equivalents) and divided on 365.25 days to calculate the mean daily doses per year. For gabapentinoids, the number of DDDs of gabapentin and pregabalin, respectively, were summarized per calendar year, and further, converted to milligrams by using WHOs standards [21]. The total dispensed doses of pregabalin and gabapentin were divided similarly on 365.25 days to calculate the mean daily dose per year. For OAT opioids, the number of DDDs of any defined OAT opioid were summarized and divided this by the number of days between the date of the first and the last dispensation per year. Due to this estimation, all patients that only were dispensed one dispensation of an OAT opioid per year were censored.

Moreover, patients were stratified into different categories according to the number of dispensations and the mean daily doses of dispensed benzodiazepines and z-hypnotics, gabapentinoids, and OAT opioids, respectively. Benzodiazepines and z-hypnotics, and gabapentinoids had three dispensation groups: 0, 1–2, and ≥ 3 dispensations per calendar year, and the OAT opioids were categorized into four groups: 1–6, 7–12, 13–51, and ≥ 52 dispensations per calendar year. Benzodiazepines and z-hypnotics were divided into three groups according to the mean daily doses (in milligrams) were dispensed: 0, ≤20, > 20 - ≤40, and > 40 diazepam equivalents. The mean daily doses of pregabalin and gabapentin were categorized into three groups. For pregabalin (mg per day): > 0 - ≤300, > 300 - ≤600, and > 600, and for gabapentin (mg per day): > 0 - ≤900, > 900 - ≤3600, > 3600. OAT opioids were defined as the mean daily DDDs, and the following three groups were used: 0- < 1, 1- < 2, and ≥ 2 mean DDDs per day. The ratio between DDD and milligrams are presented in Additional file 3.

#### Analysis strategy according to the aims

Dispensation rates were defined as all OAT patients who were dispensed one or more of the defined benzodiazepines or z-hypnotics, or gabapentinoids, respectively, per calendar year divided on all included OAT patients the same year.

Diazepam equivalents were used to adjust for equipotency of benzodiazepines and z-hypnotics. Due to the absence of consistent international guidelines of equipotency for these drugs, a sensitivity analysis was conducted [22]. The lowest and highest stated equipotency for each benzodiazepine and z-hypnotic, as well as a mean of them, respectively, were used to create three equipotency equations to convert all dispensed doses per year of each benzodiazepine or z-hypnotic into diazepam equivalents. The mean equivalent equation was as follow (in milligrams (mg)):

$$ {Diazepam\ equivalents}_{mean}= diazepam+ oxazepam\times \frac{1}{4}+ alprazolam\times \frac{40}{3}+ clonazepam\times \frac{40}{3}+ flunitrazepam\times \frac{40}{3}+ nitrazepam+ zopiclon\times \frac{8}{7}+ zolpidem\times \frac{2}{3} $$

The lowest and the highest equivalent equations are presented in Additional file 4.

The number of dispensations of benzodiazepines and z-hypnotics, gabapentinoids, and OAT opioids, respectively, was plotted against the number of dispensations of an OAT opioid per year. Furthermore, the mean daily doses of dispensed benzodiazepines and z-hypnotics, and gabapentinoids, respectively, were plotted against mean daily DDD of OAT opioids per year.

The associations between being dispensed a benzodiazepine or z-hypnotic, or a gabapentinoid, and age, gender, type of OAT opioid, the number of dispensed OAT opioids, and being dispensed a non-OAT opioid were assessed per calendar year by using logistic regression models.

Dispensation rates and the mean daily doses of benzodiazepines and z-hypnotics, and gabapentinoids were evaluated for patients who discontinued OAT. For the baseline, the dispensation rates and the mean daily doses of dispensed benzodiazepines or z-hypnotics (stated in diazepam equivalents), and gabapentinoids, respectively, were calculated for the period 180 to 90 days before discontinuation date. Furthermore, the dispensation rates and the mean daily doses of benzodiazepines or z-hypnotics, and gabapentinoids, during the last 90 days before and the 90 days after discontinuation date, respectively, were summarized separately and compared to dispensation rates and mean daily doses at baseline.

#### Statistical analyses

Means, median, percentiles, percentage, 95% confidence interval (CI), odds ratio (OR), and *p*-value are presented when appropriate. The one-sample t-test was used to calculate mean daily doses of dispensed benzodiazepines or z-hypnotics, or gabapentinoids with 95% CI. The paired sample t-test was used to compare the differences in the mean daily dose of benzodiazepines or z-hypnotics and gabapentinoids, respectively, per year among OAT patients who discontinued OAT. Multivariable analyses for categorical variables were performed per year by creating logistic regression models. For these models, being dispensed a gabapentinoid, or a benzodiazepine or z-hypnotic were dependent variables, respectively, per year. Age groups, gender, type of OAT opioid, the number of dispensed OAT opioids, and being dispensed a non-OAT opioid were independent variables and defined categorically. In addition, being dispensed a gabapentinoid was used as an independent and categorical variable when being dispensed a benzodiazepine, or a z-hypnotic was defined as a dependent variable, and vice versa. The level of statistical significance was *p* < 0.05. All patients were excluded from the calendar year they died. SPSS version 24 was used for all analyses.

### Ethical considerations

The Regional Committee for Medical and Health Research Ethics, REC vest, Norway, has approved the use of registry data for the study (approval number 2018/939/REK Vest, June 19, 2018). No informed consent from included patients was necessary.

## Results

### Basic characteristics

A total of 10,371 patients were dispensed at least one OAT opioid from pharmacies in Norway in the period 2013 to 2017 (Table 1). In 2017, 69% were men. The mean age increased from 43 (standard deviation (SD): 10) years in 2013 to 45 (SD: 10) years in 2017. A total of 690 participants died during the study period.

**Table 1:** Baseline characteristics of patients on opioid agonist therapy in Norway

Baseline characteristics	2013		2014		2015		2016		2017	
	*No.*	*%*	*No.*	*%*	*No.*	*%*	*No.*	*%*	*No.*	*%*
*Patients*	7709		7914		7958		7804		7709	
*Deaths*	165		151		138		114		124	
*Patients, excl. deaths*	7544		7763		7820		7690		7585	
*Age*										
- ≤ 25	211	3	185	2	171	2	135	2	120	2
- 26-35	1590	21	1570	20	1551	20	1403	18	1333	18
- 36-45	2724	36	2730	35	2605	33	2508	33	3292	32
- 46-55	2283	30	2449	32	2544	33	2540	33	2548	34
- ≥ 56	736	10	829	11	949	12	1104	14	1192	16
*Mean (SD)*	*43 (10)*		*44 (10)*		*44 (10)*		*44 (10)*		*45 (10)*	
*Gender*										
Men	5221	69	5390	69	5430	69	5354	70	5245	69
Women	2323	31	2373	31	2390	31	2336	30	2340	31
*OAT opioids*^a^										
Methadone, included levomethadone	3406	45	3264	42	3216	41	3066	40	2981	39
Buprenorphine^b^	4138	55	4499	58	4604	59	4624	60	4604	61
Potentially addictive drugs										
Dispensed a benzodiazepine and z-hypnotic^c^	3747	50	3809	49	3714	47	3758	49	3762	50
Dispensed a gabapentinoid	708	9	662	9	717	9	762	10	845	11

*NorPD* Norwegian Prescription Database, *OAT* Opioid agonist therapy, *SD* Standard deviation

^a^The last type of dispensed OAT opioid per year

^b^Include buprenorphine-naloxone

^c^Z-hypnotic includes zolpiclone and zolpidem

### Dispensation rates and mean daily doses of dispensed benzodiazepines, z-hypnotics, and gabapentinoids

**Fig. 1:**
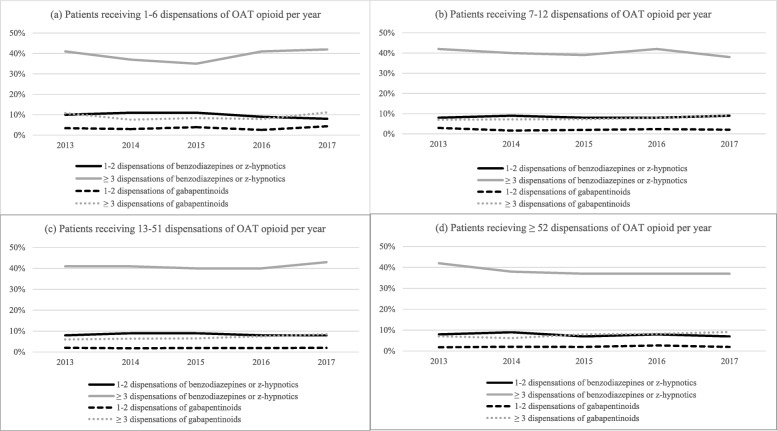
The proportion of patients on OAT were dispensed a benzodiazepine or z-hypnotic, and a gabapentinoid. Legend: OAT = opioid agonist therapy. The figures display the proportion of patients who were dispensed 1–2 or 3 or more dispensations of benzodiazepines or z-hypnotics and gabapentinoids, respectively, of those who dispensed an OAT opioid per calendar year in the study period. Each figure **a**) to **d**) displays patients on OAT categorized on the number of dispensations of OAT opioids per year: **a**) 1–6 dispensations, **b**) 7–12 dispensations, **c**) 13–51 dispensations, and **d**) 52 or more dispensations

The proportion of patients who received at least one dispensation of benzodiazepines or z-hypnotics was 50% in 2017, and 42% received three or more such dispensations (Fig. 1). Similar findings were found yearly from 2013 to 2016. The dispensation rates of benzodiazepines and z-hypnotics declined from 21 mg (95% CI: 20–23 mg) diazepam equivalents per day in 2013 to 17 mg (95% CI: 16–17 mg) diazepam equivalents per day in 2017 (Table 2a and Table 2b). A quarter of patients was dispensed oxazepam, which was the most frequently dispensed benzodiazepine per year throughout the study period (Table 3). Zopiclone was the most frequently dispensed z-hypnotic per patient per year.

**Table 2:** The daily doses of dispensed potentially addictive drugs

a)						
**All indications**		**2013**	**2014**	**2015**	**2016**	**2017**
Benzodiazepine and z-hypnotic dose per patient (in diazepam equivalents)		**mean (lowest-highest)**				
	Mean (mg/day)	21 (17–29)	20 (16–27)	19 (15–25)	17 (14–23)	17 (14–22)
	Median (mg/day)	10 (9–12)	10 (9–12)	10 (8–12)	10 (8–12)	10 (8–11)
	25 percentile (mg/day)	3 (2–3)	2 (2–3)	3 (2–3)	3 (2–3)	3 (2–3)
	75 percentile (mg/day)	23 (20–29)	22 (19–27)	21 (18–26)	21 (18–26)	20 (18–25)
**All indications**		**2013**	**2014**	**2015**	**2016**	**2017**
Pregabalin dose per patient	Mean (mg/day)	365	365	371	381	386
	Median (mg/day)	205	230	249	285	255
	25 percentile (mg/day)	46	74	62	84	69
	75 percentile (mg/day)	506	552	552	561	552
**All indications**		**2013**	**2014**	**2015**	**2016**	**2017**
Gabapentin dose per patient	Mean (mg/day)	911	970	997	960	1047
	Median (mg/day)	411	488	493	493	513
	25 percentile (mg/day)	82	124	82	82	164
	75 percentile (mg/day)	1228	1314	1383	1286	1430
b)						
**All indications**		**2013**	**2017**			
		Mean dose (95 % CI)	Mean dose (95% CI)			
	**Diazepam equivalents per patient**					
Benzodiazepine and z-hypnotic dose per patient (in diazepam equivalents)	Mean (mg/day)	21 (20–23)^a^	17 (16–17)^b^			
Pregabalin dose per patient	Mean (mg/day)	365 (309–421)^c^	386 (349–423)^d^			
Gabapentin dose per patient	Mean (mg/day)	911 (753–1068)^e^	1047 (885–1209)^f^			

*Df* Degrees of freedom, *Lowest* Lowest equipotency dose, *Highest* Highest equipotency dose, and Mean = Min + Max divided by 2

^a^One sample t-test, df = 3746, ^b^one sample t-test, df = 3761, ^c^one sample t-test, df = 486, ^d^one sample t-test, df = 590, ^e^one sample t-test, df = 260, and ^f^one sample t-test, df = 309

The table a) displays the daily doses (mean, median, 25 percentile, and 75 percentile) of dispensed benzodiazepines or z-hypnotics (calculated in diazepam equivalents), pregabalin and gabapentin per year among patients on OAT in Norway in period 2013 to 2017

The table b) displays the mean daily doses of dispensed benzodiazepines or z-hypnotics (calculated in diazepam equivalents), pregabalin and gabapentin in 2013 and 2017. The 95% confidence intervals were calculated by one-sample t-test analyses

For table a) and b), an equipotency table for benzodiazepines and z-hypnotics were used to make sensitivity analyses displaying the lowest equipotency dose and the highest equipotency dose of the included benzodiazepines and z-hypnotics. The results were presented in parentheses. All dispensed benzodiazepines and z-hypnotics were summarized per year

**Table 3:** The proportion of patients who were dispensed benzodiazepines, z-hypnotics, and gabapentinoids, respectively

Year	2013		2014		2015		2016		2017	
**Benzodiazepines or z-hypnotics**	**No.**	**%**^**a**^	**No.**	**%**^**a**^	**No.**	**%**^**a**^	**No.**	**%**^**a**^	**No.**	**%**^**a**^
Oxazepam	2094	28	2091	27	2124	27	2183	28	2229	29
Diazepam	1216	16	1251	16	1222	16	1272	17	1289	17
Zopiclone	1026	14	964	12	932	12	936	12	863	11
Nitrazepam	757	10	759	10	747	10	737	10	701	9
Clonazepam	479	6	432	6	362	5	331	4	268	4
Zolpidem	351	5	359	5	333	4	315	4	327	4
Alprazolam	305	4	304	4	237	3	206	3	196	3
Flunitrazepam	41	1	38	0	37	0	32	0	36	0
**Gabapentinoids**	**No.**	**%**^**a**^	**No.**	**%**^**a**^	**No.**	**%**^**a**^	**No.**	**%**^**a**^	**No.**	**%**^**a**^
Pregabalin	487	6	449	6	491	6	523	7	591	8
Gabapentin	260	3	240	3	277	4	291	4	310	4

*No.* Number of patients, *OAT* Opioid agonist therapy

^a^Percent of all patients who were dispensed an OAT opioid

The table displays the proportion of all OAT patients who were dispensed different benzodiazepines, z-hypnotics, and gabapentinoids

Gabapentinoids were dispensed to 9 % of the patients in 2013 and 11% of the patients in 2017. Pregabalin was almost twice as frequently dispensed per patient as gabapentin per year throughout the study period. The mean daily doses of dispensed pregabalin increased with 1% per year from 365 mg (95% CI: 309–421 mg) in 2013 to 386 mg (95% CI: 349–423 mg) in 2017, and gabapentin increased with 4% per year from 911 mg (95% CI: 753–1068 mg) to 1047 mg (95% CI: 885–1209 mg) in the same period.

### The dispensation rates of benzodiazepines and z-hypnotics, and gabapentinoids related to the number of dispensations and mean daily DDD of dispensed OAT opioids

The number of dispensations and the mean daily DDD of OAT opioids was not associated with changes in dispensation rates or the number of dispensations of benzodiazepines and z-hypnotics or gabapentinoids per year in the study period (Additional files 5, 6 and 7). However, being dispensed a benzodiazepine or z-hypnotic was associated with aging 46–55 years or being above 56 years of age rather than aging below 25 years, gender women, using methadone rather than buprenorphine as OAT opioid, or being dispensed at least one dispensation of non-OAT opioids, or gabapentinoids in 2017 (Table 4, Additional file 8). Further, being dispensed a gabapentinoid was associated with being dispensed a benzodiazepine or z-hypnotic or a non-OAT opioid. Similar results were substantially found per year in the period 2013 to 2016.

**Table 4:** Logistic regression analyses of variables associated with being dispensed a benzodiazepine or z-hypnotic, and a gabapentinoid

a)				
**2017**	Dispensed a benzodiazepine or z-hypnotic			
	*N* = 3764			
	**cOR**	***p*-value**	**aOR (95% CI)**	***p*-value**
*Age*				
- ≤ 25	1.0 (ref.)		1.0 (ref.)	
- 26-35	1.0	.80	0.9 (0.6–1.4)	.76
- 36-45	1.0	.85	1.0 (0.7–1.5)	.94
- 46-55	*1.6*	*.02*	*1.7 (1.1–2.5)*	*.05*
- ≥ 56	*1.9*	*< .01*	*1.2 (1.1–1.3)*	*.01*
*Gender*				
- Men	1.0 (ref.)		1.0 (ref.)	
- Women	*1.2*	*< .01*	*1.2 (1.1–1.3)*	*< .01*
*The number of dispensations of OAT opioids*				
- ≥ 52	1.0 (ref.)		1.0 (ref.)	
- 13-51	*1.3*	*< .01*	*1.2 (1.1–1.5)*	*.01*
- 7-12	1.1	.27	1.0 (0.9–1.3)	.73
- 1-6	*1.3*	*.03*	1.1 (0.9–1.4)	.23
*OAT opioids*^a^				
- Buprenorphine (incl. combinations)	1.0 (ref.)		1.0 (ref.)	
- Methadone (incl. Levomethadone)	*1.4*	*< .01*	*1.3 (1.2–1.4)*	*< .01*
Dispensed a non-OAT opioid	*3.5*	*< .01*	*3.0 (2.6–3.5)*	*< .01*
Dispensed a gabapentinoid	*3.0*	*< .01*	*2.5 (2.1–3.0)*	*< .01*
b)				
**2017**	Dispensed a gabapentinoid			
	*N* = 845			
	**cOR**	***p*-value**	**aOR (95% CI)**	***p*-value**
*Age*				
- ≤ 25	1.0 (ref.)		1.0 (ref.)	
- 26-35	1.1	.71	1.2 (0.6–2.2)	.60
- 36-45	1.0	.90	1.1 (0.6–2.0)	.79
- 46-55	1.0	.99	0.9 (0.5–1.7)	.75
- ≥ 56	1.0	.95	0.8 (0.4–1.5)	.45
*Gender*				
- Men	1.0 (ref.)		1.0 (ref.)	
- Women	1.3	< .01	1.1 (1.0–1.3)	.16
*The number of dispensations of OAT opioids*				
- ≥ 52	1.0 (ref.)		1.0 (ref.)	
- 13-51	0.9	.62	0.9 (0.7–1.1)	.31
- 7-12	1.0	.95	0.9 (0.7–1.3)	.72
- 1-6	*1.5*	*.01*	1.2 (0.9–1.7)	.26
*OAT opioids*^a^				
- Buprenorphine (incl. combinations)	1.0 (ref.)		1.0 (ref.)	
- Methadone (incl. Levomethadone)	1.1	.46	1.1 (0.9–1.3)	.41
Dispensed a non-OAT opioid	*3.7*	*< .01*	*3.0 (2.5–3.5)*	*<.01*
Dispensed a benzodiazepine or z-hypnotic	*3.0*	*< .01*	*2.5 (2.1–3.0)*	*<.01*

*cOR* crude odds ratio, *aOR* adjusted odds ratio, *CI* Confidence interval, and *OAT* Opioid agonist therapy

^a^The last type of dispensed OAT opioid

Table a) and b) display unadjusted (crude) and adjusted odds ratio for all independent variables of patients who were dispensed at least a benzodiazepine or z-hypnotic, and a gabapentinoid, respectively, in 2017 in Norway. a) Being dispensed at least a benzodiazepine or z-hypnotic was defined as a dependent variable, and age, gender, ‘the number of dispensations of OAT opioids,’ ‘OAT opioids,’ ‘dispensed a non-OAT opioid,’ and ‘dispensed a gabapentinoid’ were defined as categorical and independent variables. b) Being dispensed a gabapentinoid was defined as a dependent variable, and age, gender, ‘the number of dispensations of OAT opioids,’ ‘OAT opioids,’ ‘dispensed a non-OAT opioid,’ and ‘dispensed a benzodiazepine or z-hypnotic’ were defined as categorical and independent variables

### Dispensation rates and mean daily doses of dispensed benzodiazepines and z-hypnotics, and gabapentinoids related to discontinuation of OAT

**Fig. 2:**
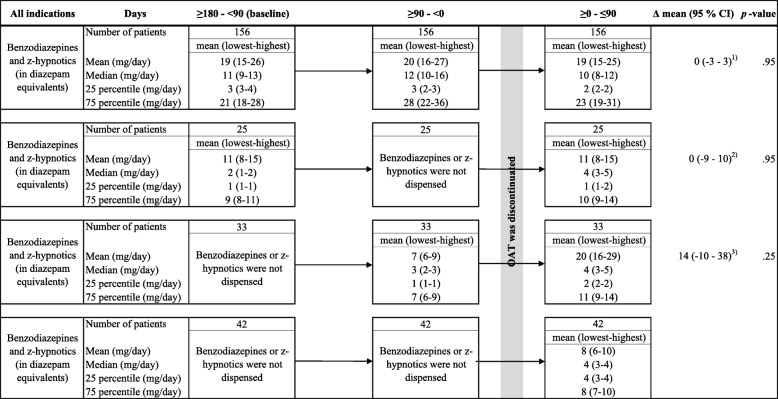
Daily doses of benzodiazepines and z-hypnotics among patients who discontinued OAT. Legends: CI = Confidence interval, df = degrees of freedom, lowest = lowest equipotency dose, highest = highest equipotency dose, and OAT = opioid agonist therapy. ^1)^ Paired t-test, df = 155, comparing mean daily dose ≥0 - ≤90 days to baseline related to discontinuation. ^2)^ Paired t-test, df = 24, comparing mean daily dose ≥0 - ≤90 days to baseline related to discontinuation. ^3)^ Paired t-test, df = 32, comparing mean daily dose ≥0 - ≤90 days to ≥90 - < 0 days related to discontinuation. Displays the daily doses of dispensed benzodiazepines and z-hypnotics, in the following period related to the date of the last dispensation of an OAT opioids: 1) 180–90 days before discontinuation (baseline), 2) 90–0 days before discontinuation, and 3) 0–90 days after discontinuation. Discontinuation was defined as all patients on OAT who had the last dispensation of an OAT opioid in the period January 1, 2017, to September 30, 2017, and no dispensation until the end of March 31, 2018. The daily doses were stated in mean, median, 25 percentile, and 75 percentile. An equipotency table for benzodiazepines and z-hypnotics were used to make sensitivity analyses, displaying the lowest equipotency dose and the highest equipotency dose of the included benzodiazepines and z-hypnotics. The results were presented in parentheses. All dispensed benzodiazepines and z-hypnotics were summarized per year

We identified 693 patients who discontinued OAT during the inclusion period. Of those, 156 patients were dispensed at least one dispensation of a benzodiazepine or z-hypnotic in the period from 180 days before to 90 days after discontinuation. The mean daily dose of dispensed benzodiazepine and z-hypnotic was not changed compared to the mean daily dose at baseline when patients discontinued OAT (Δ mean daily dose (in mg): 0, 95% CI: − 3 – 3) (Fig. 2).

Furthermore, 42 patients were dispensed a benzodiazepine or z-hypnotic only during the 90 days after discontinuation. Of these patients, the mean daily dose of benzodiazepines or z-hypnotics during the 90 days after discontinuation was about half of the mean daily dose of these patients who were dispensed benzodiazepines or z-hypnotics at baseline and until 90 days after the discontinuation. Pregabalin and gabapentin were prescribed to 50 and 23 patients, respectively, during the 180 days before to 90 days after discontinuation of OAT (Additional file 9). No changes in mean daily doses of these drugs were found when comparing the mean daily doses at baseline with the mean daily doses the first 90 days after the discontinuation (pregabalin: Δ mean daily dose (in mg): 50, 95% CI: − 47 – 149, gabapentin: Δ mean daily dose (in mg): 190 mg, 95% CI: − 789 – 1168).

## Discussion

In the period 2013 to 2017, a steady proportion of the Norwegian OAT population received at least one prescription of a benzodiazepine or z-hypnotic. Furthermore, the number of patients who were dispensed at least one dispensation of a gabapentinoid increased slightly in the study period. The mean daily dose of benzodiazepines and z-hypnotics was declining, while the mean daily dose of pregabalin and gabapentin were increasing. The number of dispensations and the mean daily DDD of OAT opioids did not affect the number of dispensations of benzodiazepines and z-hypnotics, or gabapentinoids. Being dispensed a benzodiazepine or z-hypnotic was associated with aging 46–55 years or being above 56 years of age rather than aging below 25 years, gender women, using methadone rather than buprenorphine as OAT opioid or being dispensed at least once a non-OAT opioid, or a gabapentinoid in 2017. Similar results were substantially found in the period from 2013 to 2016. Oxazepam and zopiclone were the most frequently dispensed benzodiazepine and z-hypnotic, respectively, and pregabalin was prescribed twice as often per patient per year as gabapentin throughout the study period. Discontinuation of OAT was not associated with changes in the dispensation rates or the mean daily doses of benzodiazepines or z-hypnotics, or gabapentinoids.

Our findings were in line with dispensation rates of benzodiazepines, z-hypnotics, and gabapentinoids in the OAT population in the United Kingdom in the period from 1998 to 2014 [9]. Further, the dispensation rates were higher for being dispensed a benzodiazepine or a gabapentinoid and lower for being dispensed a z-hypnotic compared with the OAT population in Sweden in the period 2005 to 2013 [1]. In Norway, the proportion of OAT patients who were dispensed benzodiazepines or z-hypnotics was at least as high as comparable descriptive analyses of benzodiazepine dispensations in 2005 [23]. Moreover, the proportion of the general Norwegian population was dispensed a benzodiazepine or z-hypnotic decreased relatively on 1.2% per year from 10.8% in 2015 to 10.4% in 2017, whereas pregabalin increased by 3.5% per year, and gabapentin increased by 5.8% per year in the same period [24]. The dispensation rates of all these potentially addictive drugs were substantially higher in the OAT population. For gabapentinoids, the dispensation rates were increasing in both the OAT population and the general Norwegian population in the study period.

The reasons for the increasing use of gabapentinoids in the OAT population are lacking. In the past decade, gabapentinoids, particularly pregabalin, were placed under scrutiny due to the risk of addiction [16], and prescribers have become aware of the risk of prescribing these drugs to patients with a history of drug addiction [1]. Although, it is remarkable that the dispensation rate of gabapentinoids was increasing, and pregabalin was dispensed twice as frequently as gabapentin. The reason may be a high prevalence of psychiatric comorbidities like anxiety in the OAT population [25–27]. Further studies evaluating reasons for the increasing gabapentinoid use among patients on OAT is required.

Being dispensed a benzodiazepine or z-hypnotic was particularly associated with being dispensed non-OAT opioids, and gabapentinoids, as well as methadone rather than buprenorphine as the type of OAT opioid. Chronic non-malignant pain like pain in muscles and skeleton is highly prevalent in the OAT population using methadone as an OAT opioid and affects up to 68% in some studies [28–31]. Having chronic non-malignant pain on OAT is strongly associated with using benzodiazepines [26, 27], and the presence of psychiatric comorbidities such as anxiety and depression [31]. Even though the prevalence of chronic non-malignant pain in the Norwegian OAT population is uncertain, one can assume that chronic non-malignant pain was an essential explanation for the association between the dispensation of benzodiazepines and gabapentinoids and using methadone on OAT in this study.

Overall, there is substantial evidence that OAT protects against overdose-related deaths and injecting opioid use [32, 33]. Nevertheless, the mortality increases significantly among patients on OAT if dispensed benzodiazepines, z-hypnotics, or gabapentinoids [1, 2]. Therefore, the guidelines in several European countries recommend careful dispensation of potentially addictive drugs to these patients on OAT [34, 35]. However, being dispensed of a potentially addictive drug is not necessarily wrong, and the reasons for these dispensations may be multifactorial. Physical and mental comorbidities are highly prevalent among patients on OAT, which predict and defend the dispensations of potentially addictive drugs [25–27]. In a few marginalized cases with several addictions, it is argued that dispensations of benzodiazepines decrease mortality if low dosed benzodiazepines replace illegal drug consumption [2]. Nevertheless, it should be a better awareness of whether such dispensations are medically indicated on patients on OAT taken our findings into consideration. Improving prescription routines among general physicians, application of strict monitoring systems, and a close co-operation with specialized addiction health care center may be considered as some of the essential approaches to strive for optimal conditions in cases on OAT where several potentially addictive drugs are medically indicated.

## Strengths and limitations

Using Norwegian registry data had some strengths. Pharmacy records are viewed to be more valid than both medical records and data collected from questionnaires and interviews. Because practically all dispensed drugs are registered in the database, completeness, and precision of all received information is high, and the potential for information biases is low.

This study also had some limitations. The NorPD only receives information about dispensed drugs, and we cannot know whether the drugs have been consumed. Second, due to that a minor part of reimbursed prescriptions being received through the Norwegian Health Economics Administration (HELFO), the medical indications for these dispensations are not available for the researchers through NorPD. For example, clonazepam, pregabalin, and gabapentin may be dispensed on the medical indication of epilepsy, while oxazepam or diazepam may be used preferably for detoxifications, or treatment of short-term anxiety or sleeping disorder. Third, the number of dispensations may be incomplete registered by the pharmacies. For OAT opioids, the self-reporting survey of OAT showed that the mean number of dispensation per patient was four times a week [15]. This finding may indicate that the number of dispensations is underestimated in our study. In order to adjust for this uncertainty to some extent, the mean daily dose calculated by summing the dispensed DDD, divided by the number of days between the first and the last dispensation were used. Fourth, slightly less than 10% of OAT opioids are dispensed in addiction specialist outpatient clinics, and those are not necessarily registered in NorPD. Some of these outpatient clinics ordered OAT opioids directly from pharmacies without linking to a personal identification number. These patients were lost in this study [15].

## Conclusion

The dispensation rates of benzodiazepines, z-hypnotics, and gabapentinoids to patients receiving OAT in Norway are high. A high burden of disease among patients on OAT may be an essential explanation. Future policies need to debate the indications for dispensations of benzodiazepines, z-hypnotics, and gabapentinoids explicitly in guidelines on OAT as well as make requirements for dispensation authority. More randomized controlled trials evaluating the benefits and risks of such co-dispensation with sufficient power are required.

## Supplementary information

**Additional file 1.** STROBE Statement. Checklist of items that should be included in reports of cohort studies.

**Additional file 2.** Anatomical Therapeutic Chemical (ATC) codes for opioids, benzodiazepines, z-hypnotics and gabapentinoids. Legends: OAT = opioid agonist therapy, and WHO = World Health Organization. * Defined by the WHO collaborating centre for drug statistics. The table displays ATC codes and the corresponding drugs defined by WHO.

**Additional file 3.** Daily defined doses (DDD) and equipotency of opioid agonist therapy (OAT) opioids, benzodiazepines and z-hypnotics, and gabapentinoids that have marketing authorizations in Norway. Legends: Lowest = Lowest equipotency dose, Highest = Highest equipotency dose.

**Additional file 4.** Equations to calculate diazepam equivalents. Legends: Equations used to calculate lowest, mean, and highest equipotency dose of included benzodiazepines and z-hypnotics.

**Additional file 5.** Dispensations of benzodiazepines or z-hypnotics, and gabapentinoids plotted against the number of dispensations of OAT opioids per year. Legends: No. = number of patients, OAT = opioid agonist therapy. Patients categorized on 0, 1–2 and ≥ 3 dispensations of benzodiazepines or z-hypnotics, and gabapentinoids per year, and plotted against the number of dispensations of OAT opioids per year in the period 2013 to 2017 in Norway.

**Additional file 6.** The mean daily DDDs of dispensed OAT opioids plotted against the mean daily doses of dispensed benzodiazepines and z-hypnotics. Legends: DDD = daily defined dose, and No. = number of patients. The table displays the mean daily DDDs of dispensed OAT opioid plotted against the mean daily doses of dispensed benzodiazepines or z-hypnotics. The number of included patients on OAT in this table were only those who have > 1 dispensation of an OAT opioid per calendar year.

**Additional file 7.** The mean daily DDD of dispensed OAT opioids plotted against the mean daily doses of dispensed pregabalin and gabapentin. Legends: DDD = daily defined dose, and No. = number of patients. The table displays the mean daily DDD of dispensed OAT opioid plotted against the mean daily doses of pregabalin and gabapentin, respectively. The number of included patients on OAT in this table were only those having > 1 dispensation of an OAT opioid per calendar year.

**Additional file 8.** Logistic regression analyses of variables associated with being dispensed a benzodiazepine or z-hypnotic, and a gabapentinoid. Legends: cOR = crude odds ratio; aOR = adjusted odds ratio; CI = confidence interval, and OAT = opioid agonist therapy. * The last type of dispensed OAT opioid. Table a) and b) display unadjusted (crude) and adjusted odds ratio for all independent variables of patients who were dispensed a benzodiazepine or z-hypnotic, and a gabapentinoid in 2017 in Norway. a) Being dispensed a benzodiazepine or z-hypnotic was defined as a dependent variable, and age, gender, ‘the number of dispensations of OAT opioids,’ ‘OAT opioids,’ ‘dispensed a non-OAT opioid,’ and ‘dispensed a gabapentinoid’ were defined as categorical and independent variables. b) Being dispensed a gabapentinoid was defined as a dependent variable, and age, gender, ‘the number of dispensations of OAT opioids,’ ‘OAT opioids,’ ‘dispensed a non-OAT opioid,’ and ‘dispensed a benzodiazepine or z-hypnotic’ were defined as categorical and independent variables.

**Additional file 9.** Changes in the mean daily doses of gabapentinoids among patients who discontinued OAT. Legends: CI = Confidence interval, df = degree of freedom, and OAT = opioid agonist therapy. ^1)^ Paired samples t-test, df = 25, comparing mean daily dose ≥0 - ≤90 days to baseline related to discontinuation. ^2)^ Paired samples t-test, df = 6, comparing mean daily dose ≥0 - ≤90 days to baseline related to discontinuation. ^3)^ Paired samples t-test, df = 6, comparing mean daily dose ≥0 - ≤90 days to ≥90 - < 0 days related to discontinuation. The tables a) and b) display the daily doses of dispensed pregabalin and gabapentin, respectively, in the following period related to the date of the last dispensation of an OAT opioids: 1) 180–90 days before discontinuation (baseline), 2) 90–0 days before discontinuation, and 3) 0–90 days after discontinuation. Discontinuation was defined as all patients on OAT who had the last dispensation of an OAT opioid in the period January 1, 2017, to September 30, 2017, and no dispensation until the end of March 31, 2018. The daily doses were stated in mean, median, 25 percentile, and 75 percentile.

## Data Availability

Supplemental tables and data sources in this observational study are available in this published article and its additional files.

## Abbreviations

AOR: Adjusted odds ratio
ATC: Anatomical Therapeutic Chemical
CI: Confidence interval
DDD: Defined Daily Doses
HELFO: The Norwegian Health Economics Administration
NorPD: The Norwegian Prescription Database
OAT: Opioid agonist therapy
OR: Odds ratio
WHO: World Health Organization
