# Supplementary material for: Dispensations of benzodiazepines, z-hypnotics, and gabapentinoids to patients receiving opioid agonist therapy; a prospective cohort study in Norway from 2013 to 2017
Source: BMC Health Serv Res. 2020 Apr 25;20:352. doi: 10.1186/s12913-020-05195-5 (PMC7183604; doi:10.1186/s12913-020-05195-5)
Supplement: Supplementary file 4 — Additional file 4. Equations to calculate diazepam equivalents. Legends: Equations used to calculate lowest, mean, and highest equipotency dose of included benzodiazepines and z-hypnotics. [file 12913_2020_5195_MOESM4_ESM.pdf]

Additional file 4

$$Diazepam\ equivalents_{\ mean}$$

$$= diazepam + oxazepam\ \times \frac{1}{4} + alprazolam \times \frac{40}{3} + clonazepam \times \frac{40}{3} + flunitrazepam\ \times \frac{40}{3} + nitrazepam + zopiclone \times \frac{8}{7} + zolpidem \times \frac{2}{3}$$

$$Diazepam\ equivalents_{\ lowest}$$

$$= diazepam + oxazepam\ \times \frac{1}{5} + alprazolam \times 10 + clonazepam \times 10 + flunitrazepam\ \times 10 + nitrazepam + zopiclone + zolpidem \times \frac{1}{2}$$

$$Diazepam\ equivalents_{\ highest}$$

$$= diazepam + oxazepam\ \times \frac{1}{3} + alprazolam \times 20 + clonazepam \times 20 + flunitrazepam \times 20 + nitrazepam + zopiclone \times \frac{4}{3} + zolpidem$$
